# Realistic threats escalating societal conflict: electoral distrust, strong leader support, and divided justice in South Korea’s 2024–2025 political crisis

**DOI:** 10.3389/fpsyg.2026.1808513

**Published:** 2026-05-04

**Authors:** Yeongjin Yu, Taeyun Jung

**Affiliations:** Social and Cultural Psychology Lab, Department of Psychology, Chung-Ang University, Seoul, Republic of Korea

**Keywords:** belief in electoral fraud, justice theory, realistic threat, strong leader support, subjectivity of justice

## Abstract

Across contemporary democracies, intensifying political polarization, electoral distrust, and rising support for strong leaders have raised concerns about democratic stability. Drawing on Intergroup Threat Theory and Justice Theory, this study examines how perceived outgroup threats relate to justice perceptions during the 2024–2025 political crisis in South Korea. Survey data from the South Korean general public collected immediately after the impeachment of President Yoon Suk Yeol show that perceptions of China as a realistic—but not symbolic—threat were associated with belief in electoral fraud, stronger support for President Yoon, and polarized justice evaluations of martial law and impeachment. Supporters viewed martial law as justified and impeachment as unjust, whereas opponents showed the opposite pattern, even after controlling for established ideological attitudes. The findings suggest that realistic threat perceptions are associated with political interpretations that prioritize ingroup security over justice concerns, as well as stronger support for strong leaders and exceptional political measures. These results show that perceived realistic threats are associated with polarized justice evaluations, as supporters and opponents interpret the same political actions in opposing ways, extending evidence on polarization beyond Western democracies to South Korea.

## 1 Introduction

On December 3, 2024, at 10:23 p.m., President Yoon Suk Yeol declared martial law, abruptly suspending parliamentary functions. Declared 45 years after the last such instance in 1979, it was unprecedented in a democratized country with more than three decades of institutional democracy (Lee and Lee, 2025). Within 2 h and 35 min, the decree was overturned through the convergence of three developments: citizens blocked armored vehicles to hinder deployment, lawmakers aggressively entered the National Assembly—some scaling its outer walls—to vote on lifting martial law, and soldiers hesitated to enforce orders. The National Assembly submitted an impeachment petition on December 14, and the Constitutional Court unanimously removed Yoon on April 4 of the following year (see Song and Choi, 2025).

Since its transition from military rule in 1987, South Korea has developed into one of the most consolidated democracies outside the Western world, characterized by competitive elections and repeated peaceful transfers of power. Despite this institutional maturity, however, South Korean politics has remained highly confrontational. Political competition is frequently shaped by intense ideological disputes over security policy and relations with regional powers, periodically producing political crises and deep societal polarization. These tensions have been further amplified by public concern about foreign influence—particularly involving China—rooted in historical and geopolitical factors associated with the legacy of the Korean War. When intertwined with persistent domestic political conflict, perceived external threats can foster support for exceptional and disruptive measures implemented by strong leadership even within mature democratic systems. In this respect, the South Korean case reflects broader dynamics observed in Western democracies, where external threat narratives have increasingly challenged democratic stability.

From a social psychological perspective, a defining feature of South Korea’s political crisis was the stark polarization in judgments about the justice of martial law and impeachment. The National Assembly argued in court that the declaration was procedurally unjust and warranted punishment for Yoon, his aides, and the military generals involved, whereas Yoon’s lawyers contended that the impeachment was procedurally flawed and that those responsible for election manipulation deserved punishment. Despite the Constitutional Court’s unanimous ruling for the National Assembly, public opinion remained deeply divided throughout the winter, escalating severe political polarization (Lee R., 2025). Such polarization extended beyond institutional arenas into the public sphere, where individuals advanced competing interpretations of the same events, indicating that judgments of procedural and retributive justice were not shared but socially contested.

The contrasting interpretations of martial law and impeachment raise the core question addressed in this study: Are perceptions of justice subjective, and what factors are associated with divergent justice judgments of the same sociopolitical event? In South Korea, justice and fairness have become central societal concerns over the past decade (see Choe, 2019; Kwak, 2022). Yet when perceptions of justice diverge, what one group views as just may be seen as unjust by another. Under these conditions, appeals to fairness may be ineffective and even exacerbate social conflict (see Mikula and Wenzel, 2000).

The second research question examines the psychological sources of justice perceptions, focusing on support for Yoon as a strong leader. Support for strong leaders has received sustained attention across the social sciences (e.g., Chong and Gradstein, 2018; Schneiker, 2020) and has become increasingly salient in social psychology amid global democratic strain (e.g., Gøtzsche-Astrup and Hogg, 2024; Ionescu et al., 2025; Pettigrew, 2017; van den Bos, 2020). During Yoon’s impeachment, supporters stormed a district court and damaged public property on January 19, 2025 (Lee, 2025b), paralleling the U.S. Capitol riot by Trump supporters (Lulat, 2023; Warf, 2023) and illustrating how strong-leader support can escalate into threats to democratic institutions across nations. Building on this context, we posit that perceived outgroup threat is a key psychological driver of such support. By integrating intergroup threat theory with justice research, this study situates South Korea’s crisis within a broader global pattern of societal conflict and democratic vulnerability.

### 1.1 Justice and its subjectivity

Justice, a means of resolving societal conflicts, is commonly divided into distinct domains (Tyler, 2012). One key domain is procedural justice, which concerns the legitimacy of decision-making processes and individuals’ rights in contexts such as martial law or impeachment. Procedural justice centers on individuals’ capacity to participate in or influence important group decisions (Lind and Tyler, 1988). Being able to voice opinions in neutral settings (Blader and Tyler, 2003) and to be heard by authorities (Smith et al., 1998; Tyler and Lind, 1992) strengthens group identification (de Cremer and Tyler, 2005; Huo et al., 2010; Tyler, 1989) and perceptions of procedural fairness, increasing acceptance of unfavorable outcomes (Skitka et al., 2003; van den Bos, 2005). In group contexts, perceived procedural fairness promotes voluntary cooperation (Blader and Tyler, 2009; de Cremer et al., 2005; Smith and Tyler, 1997; Tyler and Blader, 2003; Tyler and Jackson, 2014) and rule compliance (de Cremer and van Dijke, 2009; O’Brien and Tyler, 2019; Tyler, 2011). Despite extensive research in criminal justice (see Tyler et al., 2015) and organizational settings (for a review, see Bobocel and Gosse, 2015), procedural justice during sociopolitical events remains understudied, though perceived unfairness by police during mass protests has been linked to greater aggression (Tyler et al., 2018; see also van den Bos, 2020).

Retributive justice was another salient dimension of South Korea’s political crisis. Yoon’s martial law declaration threatened punishment of striking medical staff, and the National Assembly’s initial impeachment motion called for punishment of Yoon and his allies (Lee, 2025a). Retributive justice involves the motivation to punish deliberate violations of group rules and norms (Darley and Pittman, 2003). Such violations increase offenders’ power while rendering compliant members vulnerable (Carlsmith et al., 2002) and undermine shared values within the group (Okimoto et al., 2009; Wenzel et al., 2008). Accordingly, punishment should both curb illegitimate power and facilitate reintegration through reaffirmation of shared values (Okimoto and Wenzel, 2009). Although prior research has focused largely on punishment motivation (e.g., Bastian et al., 2013; Carlsmith et al., 2002; Okimoto et al., 2011), perceived injustice arising from past punitive actions can also provoke further punitive motivations (Tetlock et al., 2007). Experiences of retributive justice in sociopolitical events, which typically involve completed or ongoing punitive processes, therefore fall within existing punishment frameworks.

Some research examines individuals’ overall sense of justice rather than distinguishing among specific justice domains. Studies in organizational (e.g., Ambrose and Schminke, 2009; Cropanzano et al., 2001) and university contexts (e.g., Di Battista et al., 2014) show that justice is often experienced as a unified evaluation. Thus, Koreans encountering news of martial law and impeachment may judge these events as just or unjust without differentiating among domains, relying on automatic rather than systematic processing (Cropanzano et al., 2001). Even when engaging in systematic processing, individuals draw on domains with sufficient available information (Qin et al., 2015) or those linked to salient identities (Skitka, 2003). Although justice domains are theoretically (Ambrose and Schminke, 2009) and empirically distinct (e.g., Reb et al., 2006; Sweeney and McFarlin, 1993), overall justice perceptions are strongly associated with procedural and retributive justice (e.g., Ambrose and Schminke, 2009; Wenzel and Okimoto, 2010).

Early justice research emphasized justice domains and social functions while giving limited attention to the subjectivity of justice perceptions. Justice theories explain why Koreans (Choe, 2019; Kwak, 2022)—and people more broadly—value justice, highlighting two functions: reducing uncertainty by fostering trust and cooperation under limited information (Lind and van den Bos, 2002; van den Bos et al., 1997, 2001) and sustaining long-term goal pursuit despite delayed rewards (Hafer, 2000; Hafer and Rubel, 2015; Laurin et al., 2011), thereby positioning justice as a core social motive (Montada, 2011). However, justice theorists have been slower to address whether consensus on justice is attainable. Strong justice motivations may intensify conflict rather than resolve it, as justice-based policies themselves can be contested (Bobocel et al., 2002), with opposing sides viewing each other as morally deficient despite shared justice concerns (Peters et al., 2004). Consequently, justice-based interventions (Brockner and Bobocel, 2024; Okimoto and Gollwitzer, 2025) may fail to achieve broad legitimacy, and persistent disagreement can generate enduring conflict (Mikula and Wenzel, 2000).

Although the subjectivity of justice perceptions has long been recognized (Konow, 2005; Montada, 2007; Tyler et al., 1997; see also Okimoto and Gollwitzer, 2025), a comprehensive framework specifying the determinants of justice judgments remains underdeveloped. Justice perceptions are shaped by cognitive and social processes, including stereotypes, attributional biases, group dynamics, social power, motivated reasoning, and political orientation. Empirical work shows that justice judgments vary with decision-making power (Lois and Riedl, 2022) and are strongly conditioned by prior political beliefs (Taber et al., 2009; Taber and Lodge, 2016). In South Korea, individuals favorable toward Yoon are more likely to perceive his declaration of martial law as just and to discount claims of procedural unfairness, selectively attending to countervailing information. While political bias provides a compelling explanation for this subjectivity (Taber and Lodge, 2016), retrospective measurement of prior beliefs is difficult following abrupt political crises. We therefore adopt an attitudinal approach, proposing that support for Yoon systematically relates to justice judgments, consistent with emerging research on political support for strong leaders.

### 1.2 People supporting strong leaders

Support for strong leaders—including figures such as Donald Trump and Vladimir Putin—has drawn considerable scholarly attention (e.g., Chong and Gradstein, 2018; Schneiker, 2020). Although strong leadership can also take left-wing forms (e.g., Ionescu et al., 2025), the present study is limited to leaders characterized by authoritarian populism, personal charisma, and hypermasculinity (see Eksi and Wood, 2019; Pinto, 2023a, 2023b; Tempest, 2016). Research has focused extensively on Trump’s hostility toward mainstream media and his reliance on alternative information sources, a pattern also evident in Yoon’s political communication (Yim, 2022). Trump’s repeated denunciation of mainstream media as “fake news” (Forscher and Kteily, 2020; Meeks, 2020; see also Tong et al., 2020) parallels Yoon’s distrust of traditional outlets and his preference for YouTube channels that propagate generalized prejudice against foreigners and marginalized groups (Park and Bateman, 2024). In addition, Yoon’s declaration of martial law has been interpreted as reflecting authoritarian tendencies, given its restrictions on journalistic freedom and suspension of legislative functions (Lee J., 2025). Despite meaningful differences between Trump and Yoon, we seek to apply insights from the literature on strong leader support to South Korea’s recent political crisis.

Numerous studies in social psychology identify right-wing authoritarianism (RWA) and social dominance orientation (SDO) as key psychological profiles of support for strong leaders (e.g., Crowson and Brandes, 2017; Dunwoody and Plane, 2019; Forscher and Kteily, 2020; MacWilliams, 2016; Womick et al., 2019). These orientations reflect stable individual differences that shape worldviews and organize sociopolitical attitudes (Duckitt et al., 2002; Duckitt and Sibley, 2009). Two recurring features of strong leadership are particularly congruent with the motivational profiles associated with RWA and SDO. First, strong leaders frequently mobilize public support by amplifying hostility toward foreign nations or cultures and by framing immigration as a threat to legal order and social stability. Such rhetoric resonates with individuals high in RWA, who are motivated to submit to authoritative leadership in order to protect the perceived moral integrity of the ingroup from threatening outgroups (Duckitt et al., 2002). Second, strong leaders often emphasize national superiority and greatness, legitimizing the pursuit of dominance, resources, and power—even at the cost of international cooperation—while promising to dismantle policies that protect socially marginalized groups. These messages closely align with the defining elements of SDO—group-based dominance and opposition to egalitarianism (Ho et al., 2012)—rendering them especially appealing to individuals high in SDO.

Since MacWilliams (2016) seminal study linking right-wing authoritarianism (RWA) to support for Donald Trump, subsequent research has repeatedly documented that Trump supporters tend to exhibit elevated levels of both RWA and social dominance orientation (SDO). Crowson and Brandes (2017), for instance, showed that individuals who favored Trump over Hillary Clinton in the 2016 U.S. presidential election were more authoritarian, traditionalist, and resistant to egalitarian values. Complementing these findings, Womick et al. (2019) observed heightened aggressive dominance toward outgroups among Trump voters, a pattern also identified in large-scale online samples (Forscher and Kteily, 2020). Beyond shaping intergroup attitudes, support for such leadership styles may have profound consequences for democratic governance. Dunwoody and Plane (2019) found that higher RWA was associated with greater acceptance of anti-democratic policies, indicating that these orientations can undermine democratic norms from within. Overall, this line of research has established individual difference variables as dominant explanatory factors in conventional accounts of support for strong leaders.

Focusing solely on individual differences overlooks key situational influences on South Korea’s political dynamics. Attitudes toward Yoon are shaped not only by psychological dispositions but also by perceived threats from outgroups. Such threat perceptions are a consistent predictor of support for strong leaders (see Dunwoody and Plane, 2019). In Trump’s case, he expanded the list of perceived “threatening” outgroups beyond those traditionally viewed as such—such as Islamic cultural nations, Russia, and China—to include Mexico and even longstanding allies like South Korea. In a similar vein, Yoon persistently portrayed China as a threat before declaring martial law and during the impeachment process (Park, 2025). Even without objective danger, subjective threat perceptions can independently drive sociopolitical attitudes (Semyonov et al., 2004). To extend the conventional approaches, the present study focuses on a sequential association in which perceived threat relates to belief in electoral fraud, which in turn is associated with support for Yoon.

### 1.3 Perceived threats to South Korea and belief in electoral fraud

To mobilize support, strong leaders frequently depict national survival as endangered by outgroups (see Gelfand and Lorente, 2021), thereby justifying restrictive border policies and discrimination against—even forcibly remove—domestic migrant populations. Trump exemplified this strategy by advancing the U.S.–Mexico border wall and pressuring universities to expel international students who opposed his Gaza policy. Support is further cultivated through narratives in which the “virtuous” populace is harmed by collusion between “evil” outgroups and political elites (Mols and Jetten, 2016). Distinct from traditional right-wing leadership, this approach fuses foreign hostility with domestic anti-elitism. In the South Korean context, Yoon consistently framed China as a central outgroup threat (Park, 2025). Mirroring Trump’s rhetoric, Yoon alleged Chinese infiltration and portrayed opposing political elites as collaborators. During the impeachment trial, he further justified martial law by invoking the military term hybrid warfare, framing China’s economic, political, and cultural influence as psychological warfare and casting domestic dissent as wartime betrayal.

Among these threat-related narratives, belief in electoral fraud represents the most direct psychological manifestation of perceived foreign interference. Belief in electoral fraud offers the clearest insight into Yoon’s view of China as an outgroup threat (for a discussion, see Bubeck and Marinov, 2017). During martial law, he ordered troops to seize a server from the National Election Commission (NEC) and publicly alleged possible Chinese election interference in his December 12 speech. This belief closely parallels Trump’s fraud claims, with Yoon’s supporters adopting similar narratives and slogans (Choe, 2025; Valerio et al., 2025). As with Trump’s allegations in the 2016 and 2020 U.S. elections—widely rejected by experts (Auerbach and Pierson, 2021; Cottrell et al., 2018; Eggers et al., 2021)—Yoon’s claims regarding South Korea’s 2020 general election were dismissed by the judiciary (see Song and Choi, 2025). Distinctively, Yoon also questioned the legitimacy of the 2022 presidential election that he won, violating a long-standing tradition among victorious candidates (Levy, 2021; Sinclair et al., 2018). Telling his aides that he had expected a landslide victory, he attributed his narrow victory to Chinese interference, which directly motivated the military intervention at the NEC. Unlike U.S. cases that emphasized Russian involvement and mail-in ballots (Office of the Director of National Intelligence, 2017; Senate Intelligence Committee, 2018), Yoon focused on China and the early voting system. Despite differences in the specific allegations involved, parallels in narratives and slogans suggest conceptual equivalence in beliefs in electoral fraud across the South Korean and the U.S. contexts.

Yoon’s tendency to attribute electoral outcomes to foreign manipulation—even following electoral victories—implies that belief in fraud is driven by psychological processes beyond political self-interest. Existing research emphasizes susceptibility to misinformation as a key mechanism (for a review, see Ecker et al., 2022). In the U.S. context, conservative orientation predicts greater receptivity to conspiracy thinking (Enders and Smallpage, 2019), which in turn fosters fraud beliefs (Edelson et al., 2017; Enders et al., 2021). Such beliefs are further reinforced by conservative media exposure and higher RWA (Borah et al., 2025), as well as difficulties in identifying misinformation within ideologically homogeneous networks (Blanchar and Norris, 2021; Calvillo et al., 2021a; Calvillo et al., 2021b). Belief in electoral fraud has been shown to increase support for economic retaliation against suspected foreign actors (Tomz and Weeks, 2020), though its implications for democratic support remain contested (Albertson and Guiler, 2020; Berlinski et al., 2023; Norris, 2019; Thomas et al., 2025).

The literature on belief in electoral fraud exhibits three key limitations. First, it has largely overlooked perceived outgroup threat. Allegations by Trump and Yoon that foreign powers—Russia and China, respectively—manipulated elections and infiltrated national economic, political, and cultural systems may increase public receptivity to fraud narratives involving hacking or misinformation (see Tomz and Weeks, 2020), yet this relationship remains empirically untested. Second, research has insufficiently examined how such beliefs contribute to social antagonism and political polarization. Although belief in electoral fraud is linked to greater trust in strong leaders (Borah et al., 2025), justice theory may clarify how this dynamic fuels polarization. Third, the heavy reliance on American samples constrains cross-cultural generalizability. Conversely, online discourse surrounding South Korea’s 21st general election fraud allegations emphasized punitive actions by investigative authorities rather than electoral procedures themselves (Lee et al., 2024), limiting the direct applicability of American-based findings.

China was a central concern in Yoon’s claims about hybrid warfare and his persistent belief in electoral fraud during the impeachment hearings. By emphasizing China’s growing economic, political, and cultural influence and alleging collusion with opposition political elites, Yoon advanced a narrative in which foreign threat was directly linked to electoral integrity. Accordingly, the present study focuses on belief in electoral fraud as the key mechanism through which perceived outgroup threat is associated with support for Yoon. To specify the nature and origins of these perceived threats, we draw on intergroup threat theory (ITT) (Stephan et al., 2015).

### 1.4 Explaining strong leader support using intergroup threat theory

Intergroup relations are generally more competitive and hostile than interpersonal relations (Wildschut et al., 2003), and people often anticipate that outgroup members will behave deceptively or competitively in everyday interactions (Wildschut et al., 2004). These expectations are shaped by factors such as intergroup anxiety, which heighten perceptions that outgroups may infiltrate or undermine the ingroup’s integrity (for a review, see Stephan, 2014). Perceived outgroup threat is conceptualized here as a cognitive appraisal, distinct from emotions or generalized attitudes. It differs from fear in that it can elicit a range of emotional responses at both individual and group levels (Stephan and Renfro, 2002), thereby fostering negative evaluations, prejudice, and intergroup bias (Riek et al., 2006). Unlike prejudice, perceived threat can be systematically manipulated in experimental settings (Rios et al., 2018; Makashvili et al., 2018). Scholars have distinguished perceived threats across contexts as either realistic, concerning immediate tangible dangers or material losses, or symbolic, concerning ingroup identity, norms, or values.

Reflecting each type of threat, Intergroup threat theory (ITT) integrates two major research traditions on perceived outgroup threat: realistic threat (LeVine and Campbell, 1972) and symbolic threat (Sears and Kinder, 1985). Realistic threat, rooted in realistic group conflict theory, explains intergroup conflicts as arising when groups with shared goals compete for limited resources. When ingroups and outgroups perceive themselves as competitors with opposing interests, negative attitudes toward the outgroup emerge (Brown et al., 2001; Esses et al., 1998; Zárate et al., 2004), reflecting a zero-sum dynamic. For example, North Americans who view migrants as competitors for employment tend to oppose migration (Esses et al., 2001), even when migrants are well assimilated (Maddux et al., 2008). Because realistic threats directly implicate immediate security, they are often associated with immediate and exceptional responses intended to neutralize the threat, sometimes even involving violent or disruptive measures (Rozmann, 2025). In South Korea, realistic threat perceptions toward China are intensified by concerns that economic cooperation increases competition with Chinese suppliers (Jung and Jeong, 2016), alongside allegations that Chinese immigrants exploit unemployment benefits (Song, 2023) and misinformation suggesting that unqualified Chinese students gain undue advantage through scholarships awarded by prestigious universities (Kim, 2025). Perceived threat is further amplified by the size of the outgroup (Schlueter and Scheepers, 2010). Given China’s vastly larger population relative to South Korea, Yoon’s framing of China as a threatening outgroup likely heightened perceptions of realistic threat.

Symbolic threat, subsequently developed within symbolic racism theory (Sears and Kinder, 1985), argues that intergroup conflict can arise from perceived differences in values, culture, or lifestyles, even without direct competition over tangible resources (Sears and Kinder, 1985). Greater perceived value divergence is consistently associated with stronger negative outgroup attitudes (Biernat et al., 1996; Dunbar et al., 2000). In migration research, symbolic threat often outweighs economic concerns, with cultural impact shaping attitudes more strongly than material competition (Hainmueller and Hopkins, 2014), particularly in evaluations of Muslim migrants (Obaidi et al., 2018; Uenal, 2016). However, we do not expect symbolic threat to play a central role in Koreans’ perceptions of China. Although negative views of China globally are often attributed to its value system and human rights practices (Silver et al., 2022), Koreans primarily identify interference in domestic affairs as the key concern and place less emphasis on human rights issues (Silver et al., 2022, p. 19). Although this report does not directly measure the two types of threats conceptualized by ITT, it still provides meaningful insights into the realistic and symbolic threats presented by China to Koreans.

We expect Koreans to perceive China as a powerful and threatening outgroup. Considering China’s proximity and population size—approximately 30 times that of South Korea—Koreans may harbor concerns about potential infringements on their resource interests (see Schlueter and Scheepers, 2010). Drawing on intergroup threat theory, we distinguish realistic threats based on resource competition from symbolic threats rooted in value conflict. In the present study, realistic threat is conceptualized specifically as perceived economic and resource-related burdens associated with Chinese immigrants rather than geopolitical threat posed by China as a state. We hypothesize that realistic threats from China are perceived as stronger than symbolic threats. Although China is frequently criticized for human rights violations (Silver et al., 2022), and symbolic threat cannot be ruled out, existing research primarily documents the consequences of realistic threat perceptions related to Chinese immigrants and economic influence (Ma and Ma, 2023; Maddux et al., 2008; Jung and Jeong, 2016; Kim, 2025; Song, 2023). Most importantly, South Korean public discourse surrounding national security and regional foreign influence frequently frames neighboring countries as posing immediate risks to national interests and sovereignty. Such narratives may therefore amplify the impact of perceived realistic threat, making it particularly influential in shaping political attitudes and perceptions of controversial sociopolitical events. Accordingly, we anticipate that perceived realistic threats—not symbolic threats—will be associated with belief in electoral fraud, which serves as a key mechanism linking threat perceptions to support for Yoon.

### 1.5 Study overview

Against the backdrop of South Korea’s declaration of martial law on December 3, 2024, and subsequent impeachment on April 4, 2025, this study examines the subjectivity of justice perception (Montada, 2007), a dimension that remains underexplored in empirical justice research. The declaration of martial law in a consolidated democracy of more than three decades represents an exceptionally rare political rupture (Lee and Lee, 2025), one that polarized Korean society into opposing camps with sharply divergent justice perceptions (Lee R., 2025). Rather than alleviating conflict, these divergences intensified and prolonged sociopolitical tensions (see Mikula and Wenzel, 2000).

**Figure 1:**
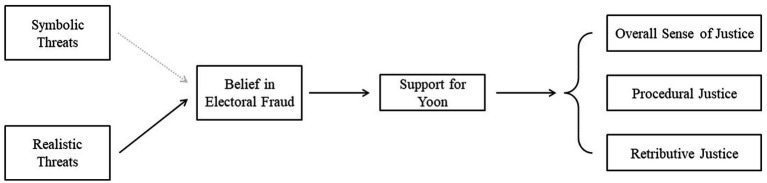
Hypothesized model linking perceived outgroup threats to belief in electoral fraud, support for Yoon, and justice perceptions.

Building on this context, we propose a sequential model linking perceived threat, political beliefs, and justice perceptions. Specifically, perceived realistic threat—conceptualized as perceived economic and resource-related burdens associated with Chinese immigrants—is expected to be associated with belief in electoral fraud, reflecting concerns about foreign interference in democratic processes. Belief in electoral fraud is expected to be associated with support for Yoon, which in turn is linked to opposing evaluations of overall, procedural, and retributive justice regarding both martial law and impeachment, such that supporters view martial law as more just and impeachment as less just, whereas opponents show the reverse pattern (see Figure 1). This reflects polarization as divergent justice judgments of the same political events across supporters and opponents. While prior research has consistently identified RWA and SDO as key predictors of support for strong leaders, these variables are treated in the present study as baseline explanatory factors rather than components of the hypothesized sequential mechanism. To assess the added value of the proposed model, we compare baseline models including RWA and SDO (Model 1) with extended models that incorporate the proposed predictors (Model 2), thereby evaluating the incremental explanatory power of perceived realistic threat and belief in electoral fraud.

The value of this study lies in the temporal proximity of data collection to Yoon’s impeachment, which strengthens internal validity by minimizing contamination from subsequent political developments. Because South Korean law mandates a presidential election within 60 days of impeachment, electoral campaigning could have rapidly reshaped public evaluations of martial law and impeachment. To reduce this threat, we collected online survey data from April 8 to 14, beginning 4 days after Yoon’s removal from office. As neither the election date nor candidates had been finalized during this period, the data capture relatively uncontaminated psychological responses to the impeachment itself.

## 2 Methods

### 2.1 Participants

We administered a survey of 800 Koreans between April 8 and 14 via Embrain Public, a South Korean online polling firm. All participants provided informed consent electronically prior to participation. The sample comprised Korean general public aged 19 to 64 years from across the country, who were able to complete the web questionnaire using a computer or smarphone ( m=400 , f=400 , $M_{\mathrm{age}}=44.06$ , $SD_{\mathrm{age}}=13.251$ ). A total of 160 respondents were evenly sampled across age groups—20s through 60–64—to minimize potential bias caused by younger, internet-proficient respondents overshadowing the perspectives of middle-aged and older adults. Respondents holding non-South Korean nationalities or occupying positions in legislative, judicial, or administrative public offices were excluded from the sample. Individuals providing identical responses across all items (i.e., straight-lining) or omitting one or more items were prompted to verify their answers by a pop-up window. Respondents who completed the survey were compensated with a participation fee below ₩1,000, in accordance with Embrain Public’s established standards.

### 2.2 Measures

All scales in the survey employed a 7-point Likert format (1 = completely disagree, 7 = completely agree), except two measures assessing support for Yoon and his foreign policy, which used endpoints defined as 1 = not at all supportive and 7 = very supportive. The phrasing of the survey items was modified to align with the South Korean sociopolitical context and the aims of our study.

*Overall sense of justice*. To measure the overall sense of justice regarding the December 3 martial law and April 4 impeachment was measured using a revised 7-item scale (Wenzel and Okimoto, 2010) adapted for sociopolitical contexts (e.g., “My feeling that people have been treated unfairly has faded.”). The scale showed high reliability for both events (martial law: α=0.869 ; impeachment: α=0.875 ). Overall, respondents perceived martial law as unjust ( M=2.27 , SD=1.371 ), and impeachment as just ( M=5.23 , SD=1.518 ).

*Procedural justice*. Perceived procedural justice for martial law and impeachment was assessed with a revised 6-item scale (Sondak and Tyler, 2012; e.g., “The procedure of the impeachment treated the people involved with dignity and respect.”). Internal consistency was high for both events (martial law: α=0.948 ; impeachment: α=0.933 ). Overall, respondents perceived martial law as procedurally unfair ( M=3.13 , SD=1.941 ), whereas they regarded impeachment as procedurally fair ( M=5.21 , SD=1.656 ).

*Retributive justice*. Retributive justice was measured using the 6-item Justice-Restoring Orientation Scale (Okimoto et al., 2011), adapted to assess perceived justice restoration following martial law and impeachment (e.g., “The declaration of martial law served as a basis for punishing offenders’ actions.”). Because martial law was rescinded within 3 h, respondents were instructed to imagine a counterfactual scenario in which it had been fully implemented. Reliability was high for both event (martial law: α=0.868 ; impeachment: α=0.802 ). Overall, respondents expressed neutrality toward the retributive justice of martial law ( M=4.07 , SD=1.755 ), whereas they regarded impeachment as retributively fair ( M=5.29 , SD=1.317 ).

*Support for Yoon*. Support for Yoon was measured in the immediate aftermath of martial law and impeachment, rather than under routine political conditions. Based on media accounts of pro-Yoon demonstrations (Choe, 2025; Valerio et al., 2025), supporters were defined as those endorsing martial law on December 3 while opposing impeachment on April 4. Accordingly, support was assessed with two items: “How much do you support the martial law declared on December 3?” and “How much do you support the Constitutional Court’s ruling on the impeachment on April 4?” (reverse-coded). The two items were strongly correlated ( r=0.690 ).

Overall, support for Yoon was minimal ( M=2.10 , SD=1.722 ). A majority of respondents (59.8%, 478 of 800) selected the lowest possible score on the 7-point scale, indicating complete opposition to martial law and strong support for impeachment. Only 75 respondents (9.4%) reported support for Yoon above the scale midpoint, resulting in a sample with substantially more opponents than typically portrayed in media coverage (Choe, 2025; Valerio et al., 2025).

*Support for Yoon’s foreign policies*. Respondents evaluated Yoon’s foreign policy toward South Korea’s neighboring countries (the United States, Japan, China, Russia, and North Korea) using a single item: “How much do you support Yoon’s foreign policies toward these countries in general?” This measure was included to capture the broader sociopolitical context surrounding Yoon’s support base, particularly the pro-U.S. and anti-China sentiments observed in media coverages (see Kim, 2024; Choe, 2025; Valerio et al., 2025). To provide descriptive context, respondents also indicated their support for closer relations with each of these countries. Descriptive statistics and correlations among these variables are reported in the Supplementary materials. These measures are included as contextual indicators of the broader sociopolitical environment (e.g., pro-U.S. and anti-China sentiments) but are not incorporated into the hypothesized model, as doing so would introduce conceptual redundancy with leader support and risk circular explanations.

*Belief in electoral fraud*. Belief in electoral fraud was measured using the 4-item Election Conspiracy Scale (Thomas et al., 2025), which assesses agreement with claims that hidden forces manipulated election outcomes. Items were adapted to South Korea’s early voting system for contextual relevance (e.g., “In the last general and presidential elections, the legitimate process of early voting was being interfered with by hidden forces.”). Although the scale was originally developed in the U.S. electoral context, its use here is appropriate because fraud narratives during South Korea’s 2024–2025 political crisis closely paralleled those observed in the United States (Choe, 2025; Valerio et al., 2025), indicating strong conceptual equivalence in beliefs about electoral illegitimacy. While the specific allegations differed, the underlying belief structure remained comparable across the two contexts. In addition, belief in electoral fraud is conceptually distinct from support for Yoon, as individuals may endorse electoral distrust while opposing the leader. The scale demonstrated excellent reliability ( α=0.952 ). Overall, respondents exhibited low endorsement of belief in electoral fraud ( M=2.98 , SD=1.945 ).

*Symbolic and realistic threat*. Perceived symbolic and realistic threats from Chinese immigrants were measured using an ITT-based scale (Stephan et al., 1999). The symbolic threat subscale consisted of 7 items (e.g., “The values and beliefs of Chinese immigrants regarding social relations are not compatible with the beliefs and values of most Koreans.”), followed by the realistic threat subscale with 8 items (e.g., “Chinese immigration has increased the tax burden on Koreans.”). To enhance validity, respondents were instructed to recall recent media coverage about China prior to responding. This instruction was intended to provide a concrete context for evaluating perceived intergroup threat, thereby reducing potential confusion between threat perceptions and generalized prejudice toward the outgroup (Makashvili et al., 2018), without presenting specific evaluative information about China. Both scales showed good reliability (symbolic threat: α=0.823 ; realistic threat: α=0.835 ), and were strongly correlated ( r=0.702 ). Overall, respondents reported experiencing moderate levels of both symbolic ( M=4.66 , SD=1.126 ) and realistic threats ( M=4.54 , SD=1.107 ) from Chinese immigrants.

*Right-wing authoritarianism*. RWA was measured using a 7-item Authoritarian Submission and Aggression subscale adapted from Manganelli Rattazzi et al.’s (2007) measure. Items inappropriate for the Korean sociopolitical context were abbreviated (e.g., “We have to crack down harder on deviant groups and troublemakers, if we are going to save our moral standards and preserve law and order.”). The scale demonstrated good reliability ( α=0.849 ). Overall, respondents exhibited moderate levels of RWA ( M=3.82 , SD=1.340 ).

*Social dominance orientation*. SDO was measured using the 8-item SDO_7(s)_ scale (Ho et al., 2015), assessing group-based dominance and opposition to egalitarianism (e.g., “An ideal society requires some groups to be on top and others to be on the bottom.”; “Group equality should not be our primary goal.”). The scale showed acceptable reliability ( α=0.749 ). Overall, respondents exhibited relatively lower levels of SDO ( M=3.03 , SD=1.013 ).

### 2.3 Procedure

Respondents were recruited using disproportionate stratified sampling by gender and age, reported at survey entry. Following consent, participants indicated their support for the December 2024 martial law and the April 2025 impeachment. Justice perceptions were measured using validated scales of overall, procedural, and retributive justice (Okimoto et al., 2011; Sondak and Tyler, 2012; Wenzel and Okimoto, 2010), administered for impeachment and then martial law. Subsequent measures assessed belief in electoral fraud (Thomas et al., 2025), support for Yoon’s foreign policies, foreign relations attitudes, symbolic and realistic threat from Chinese migrants (Stephan et al., 1999), and individual differences in RWA and SDO (Ho et al., 2015; Manganelli Rattazzi et al., 2007). The survey took approximately 10–20 min to complete.

## 3 Results

**Figure 2:**
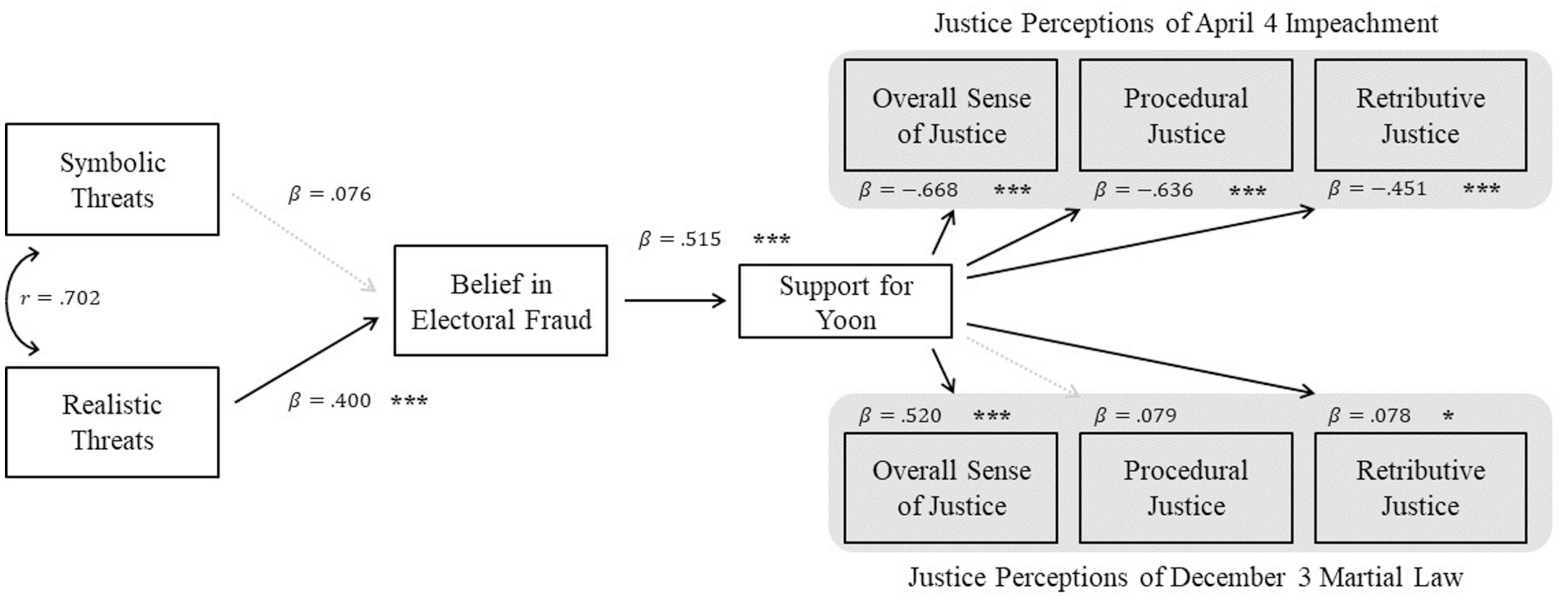
Estimated standardized path coefficients ( β ) or Pearson’s r values for the proposed model. Solid arrows represent significant effects ( p<0.05 ), and dashed arrows represent nonsignificant effects. Significance levels are indicated by asterisks (* p<0.05 , *** p<0.001 ).

Figure 2 presents the estimated path model and shows standardized coefficients for the hypothesized associations among variables. The model reflects sequential relationships from perceived threat to belief in electoral fraud, support for Yoon, and justice evaluations. These analyses were conducted as a path model examining direct associations, and indirect effects were not formally tested.

Descriptive statistics and correlations for foreign policy–related variables are reported in the Supplementary materials, including support for closer relations with South Korea’s neighboring countries. Consistent with the broader political context, support for Yoon’s foreign policy was positively associated with favorable attitudes toward the United States and Japan and negatively associated with attitudes toward China, Russia, and North Korea. Overall, respondents expressed a negative attitude toward Yoon’s foreign policies ( M=2.80 , SD=1.858 ) (Table 1).

**Table 1:** Regression results including unstandardized coefficients, *t*-values, and *p*-values for each predictor across two models: Model 1, which includes only RWA and SDO, and Model 2, which incorporates additional theoretical predictors.

Predictor	*Model 1*			*Model 2*		
	beta	*t*–value	*p*–value	beta	*t*–value	*p*–value
A. Martial law (Dec 3)—overall sense of justice						
Support for Yoon	–	–	–	0.520	24.757	<0.001***
RWA	0.342	9.605	<0.001***	0.087	3.029	0.003**
SDO	0.294	6.230	<0.001***	0.174	4.875	<0.001***
B. Martial law—procedural justice						
Support for Yoon	–	–	–	0.079	1.821	0.069
RWA	0.357	6.456	<0.001***	0.318	5.381	<0.001***
SDO	0.043	0.586	0.558	0.025	0.337	0.736
C. Martial law—retributive justice						
Support for Yoon	–	–	–	0.078	2.003	0.045*
RWA	0.370	7.424	<0.001***	0.332	6.222	<0.001***
SDO	−0.087	−1.324	0.186	−0.105	−1.585	0.113
D. Impeachment (April 4)—overall sense of justice						
Support for Yoon	–	–	–	−0.668	−36.416	<0.001***
RWA	−0.507	−13.282	<0.001***	−0.179	−7.150	<0.001***
SDO	−0.204	−4.033	<0.001***	−0.051	−1.618	0.106
E. Impeachment—procedural justice						
Support for Yoon	–	–	–	−0.636	−23.702	<0.001***
RWA	−0.425	−9.519	<0.001***	−0.113	−3.084	0.002**
SDO	−0.181	−3.072	0.002**	−0.036	−0.782	0.435
F. Impeachment—retributive justice						
Support for Yoon	–	–	–	−0.451	−19.145	<0.001***
RWA	−0.236	−6.502	<0.001***	−0.015	−0.454	0.650
SDO	−0.236	−4.921	<0.001***	−0.133	−3.311	0.001**
G. Support for Yoon						
Belief in Electoral Fraud	–	–	–	0.253	10.590	<0.001***
RWA	0.490	10.862	<0.001***	0.098	2.861	0.004**
SDO	0.229	3.836	<0.001***	−0.031	−0.737	0.461
H. Belief in electoral fraud						
Symbolic Threat	–	–	–	0.076	1.009	0.313
Realistic Threat	–	–	–	0.400	5.252	<0.001***
RWA	0.569	11.145	<0.001***	0.438	8.341	<0.001***
SDO	0.218	3.229	0.001**	0.176	2.687	0.007**

* p<0.050 ** p<0.010 *** p<0.001 . RWA, right-wing authoritarianism; SDO, social dominance orientation.

### 3.1 Subjectivity of justice and support for Yoon

*Overall sense of justice of martial law*. To examine the association between support for Yoon and overall justice perceptions of martial law, we conducted hierarchical regression analyses comparing baseline models including RWA and SDO (Model 1) with extended models incorporating support for Yoon (Model 2). The inclusion of support for Yoon significantly improved explanatory power, increasing adjusted $R^{2}=0.559$ ( $\Delta R^{2}=0.338$ ), $F_{\left(3,796\right)}=339.127$ , p<0.001 . Support for Yoon was significantly associated with the overall sense of justice regarding martial law ( β=0.520 , $t_{\left(799\right)}=24.757$ , p<0.001 ), and both RWA ( β=0.087 , $t_{\left(799\right)}=3.029$ , p=0.003 ) and SDO ( β=0.174 , $t_{\left(799\right)}=4.875$ , p<0.001 ) remained significant as control variables. These results indicate that support for Yoon is strongly associated with higher perceived overall justice of martial law, above and beyond individual difference variables.

*Procedural justice of martial law*. We next examined perceived procedural justice of martial law using the same modeling approach. The inclusion of support for Yoon resulted in a minimal increase in explanatory power (adjusted $R^{2}=0.066$ , $\Delta R^{2}=0.004$ ), $F_{\left(3,796\right)}=19.967$ , p<0.001 . Support for Yoon was a marginal predictor ( β=0.079 , $t_{\left(799\right)}=1.821$ , p=0.069 ), whereas RWA was a strong positive predictor ( β=0.318 , $t_{\left(799\right)}=5.381$ , p<0.001 ) and SDO was nonsignificant ( β=0.025 , $t_{\left(799\right)}=0.337$ , p=0.736 ). These findings suggest that procedural justice perceptions are more closely associated with authoritarian predispositions than with leader support.

*Retributive justice of martial law*. For perceived retributive justice of martial law, the inclusion of support for Yoon produced a modest increase in explanatory power (adjusted $R^{2}=0.071$ , $\Delta R^{2}=0.005$ ), $F_{\left(3,796\right)}=21.375$ , p<0.001 . Support for Yoon ( β=0.078 , $t_{\left(799\right)}=2.003$ , p=0.045 ) and RWA ( β=0.332 , $t_{\left(799\right)}=6.222$ , p<0.001 ) were significant predictors, whereas SDO was not ( β=−0.105 , $t_{\left(799\right)}=-1.585$ , p=0.113 ). These results indicate that retributive justice perceptions are modestly associated with both leader support and authoritarian tendencies.

*Overall sense of justice of impeachment*. We then examined overall sense of justice of impeachment. The inclusion of support for Yoon substantially increased explanatory power, with adjusted $R^{2}$ reaching 0.726 ( $\Delta R^{2}=0.455$ ), $F_{\left(3,796\right)}=706.991$ , p<0.001 . Support for Yoon was a strong negative predictor ( β=−0.668 , $t_{\left(799\right)}=-36.416$ , p<0.001 ), with RWA also significant ( β=−0.179 , $t_{\left(799\right)}=-7.150$ , p<0.001 ), whereas SDO was not ( β=−0.051 , $t_{\left(799\right)}=-1.618$ , p=0.106 ). These findings indicate that stronger support for Yoon is associated with lower perceived justice of impeachment.

*Procedural justice of impeachment*. For procedural justice of impeachment, the inclusion of support for Yoon increased explanatory power to adjusted $R^{2}=0.508$ ( $\Delta R^{2}=0.346$ ), $F_{\left(3,796\right)}=276.173$ , p<0.001 . Support for Yoon ( β=−0.6 636, $t_{\left(799\right)}=-23.702$ , p<0.001 ) and RWA ( β=−0.113 , $t_{\left(799\right)}=-3.084$ , p=0.002 ) were significant predictors, whereas SDO was not ( β=−0.036 , $t_{\left(799\right)}=-.782$ , p=0.435 ). These results suggest that perceptions of procedural injustice are strongly associated with leader support.

*Retributive justice of impeachment*. For retributive justice of impeachment, the inclusion of support for Yoon increased explanatory power to adjusted $R^{2}=0.401$ ( $\Delta R^{2}=0.275$ ), $F_{\left(3,796\right)}=179.492$ , p<0.001 . Support for Yoon ( β=−0.451 , $t_{\left(799\right)}=-19.145$ , p<0.001 ) and SDO ( β=−0.133 , $t_{\left(799\right)}=-3.311$ , p=0.001 ) were significant predictors, whereas RWA was not ( β=−0.015 , $t_{\left(799\right)}=-0.454$ , p=0.650 ). These findings indicate that retributive justice perceptions are associated with both leader support and endorsement of group-based hierarchy.

Taken together, justice perceptions of martial law and impeachment were sharply polarized by support for Yoon: supporters judged martial law as just and impeachment as unjust, whereas opponents exhibited the opposite pattern. RWA showed stronger associations with procedural and retributive justice perceptions of martial law than support for Yoon, while SDO showed minimal associations, reaching significance only for retributive justice perceptions of impeachment.

### 3.2 Belief in electoral fraud as a predictor of support for Yoon

To evaluate the proposed mechanism, hierarchical regression analyses compared baseline models including RWA and SDO (Model 1) with extended models incorporating belief in electoral fraud (Model 2). The inclusion of belief in electoral fraud significantly improved explanatory power, increasing adjusted $R^{2}=0.417$ ( $\Delta R^{2}=0.210$ ), $F_{\left(3,796\right)}=191.675$ , p<0.001 . Belief in electoral fraud emerged as a strong predictor ( β=0.515 , $t_{\left(799\right)}=16.976$ , p<0.001 ), while both RWA ( β=0.180 , $t_{\left(799\right)}=5.550$ , p<0.001 ) and SDO ( β=0.076 , $t_{\left(799\right)}=2.517$ , p=0.012 ) also contributed significantly. These results demonstrate that belief in electoral fraud substantially enhances the explanatory power of conventional models based on individual differences.

### 3.3 Perceived symbolic and realistic threats from China

Finally, we examined the role of perceived threat in shaping belief in electoral fraud. Hierarchical regression analyses compared baseline models including RWA and SDO (Model 1) with extended models incorporating symbolic and realistic threat (Model 2). The inclusion of threat variables increased explanatory power to adjusted $R^{2}=0.260$ ( $\Delta R^{2}=0.057$ ), $F_{\left(3,796\right)}=71.170$ , p<0.001 . Perceived realistic threats from China are strongly associated with belief in electoral fraud ( β=0.400 , $t_{\left(799\right)}=5.252$ , p<0.001 ), whereas symbolic threats were nonsignificant ( β=0.076 , $t_{\left(799\right)}=1.009$ , p=0.313 ). Both RWA ( β=0.438 , $t_{\left(799\right)}=8.341$ , p<0.001 ) and SDO ( β=0.176 , $t_{\left(799\right)}=2.687$ , p=0.007 ) contributed independently. These results indicate that belief in electoral fraud is associated with realistic threat perceptions as well as individual difference variables.

Additional regressions entering each threat variable separately indicated that both realistic and symbolic threat were significantly associated with belief in electoral fraud and support for Yoon’s foreign policy ( $F_{\left(3,796\right)}=94.551$ for realistic threat; $F_{\left(3,796\right)}=82.927$ for symbolic threat, ps<0.001 ). When both predictors were entered simultaneously, however, only realistic threat remained significant. Multicollinearity diagnostics indicated acceptable levels ( VIF=2.030 for realistic threat; VIF=2.050 for symbolic threat; tolerance=0.480 – 0.500 ), suggesting that the observed pattern reflects shared variance between the two threat perceptions rather than problematic multicollinearity.

## 4 Discussion

### 4.1 Implications of the major findings

Yoon’s declaration of martial law and subsequent impeachment represented rare and destabilizing events in South Korea’s consolidated democracy (Lee and Lee, 2025), contributing to acute polarization over their perceived justice (Lee R., 2025). While justice is theorized as a core human motive (Montada, 2011) and policy-relevant principle (Brockner and Bobocel, 2024), its subjectivity may amplify sociopolitical conflict rather than resolve it (Mikula and Wenzel, 2000; Montada, 2007; Okimoto and Gollwitzer, 2025). Addressing the paucity of empirical evidence from real-world crises, we demonstrated that justice perceptions of these events were systematically associated with support for Yoon, even after controlling for RWA and SDO—key predictors in prior research on strong leaders (e.g., Crowson and Brandes, 2017; Dunwoody and Plane, 2019; Forscher and Kteily, 2020; MacWilliams, 2016; Womick et al., 2019). Consistent with observations of pro-Yoon mobilization emphasizing foreign threats and electoral interference (Choe, 2025; Valerio et al., 2025), and informed by intergroup threat theory (Stephan et al., 2015), we found that perceived realistic threat from China—particularly economic and resource-related concerns—was more strongly associated than symbolic threat (e.g., Jung and Jeong, 2016; Kim, 2025; Song, 2023). To reduce confounding effects of the impending presidential election, data were collected within 10 days of impeachment.

Our first research question addressed the subjectivity of justice perceptions surrounding martial law and impeachment. Despite evaluating identical events, Koreans’ judgments of overall, procedural, and retributive justice diverged sharply as a function of support for Yoon: supporters viewed martial law as just and impeachment as unjust, whereas opponents perceived the reverse. This pattern is consistent with justice theory (Montada, 2007) and prior empirical work on subjective justice judgments (e.g., Bobocel et al., 2002; Lois and Riedl, 2022). Importantly, this polarization did not reflect asymmetries in justice motivation (see Peters et al., 2004) but rather strong and conflicting justice commitments on both sides (Mikula and Wenzel, 2000; Montada, 2011). Social psychologists have long argued that sociopolitical conflict arises not from moral or cognitive deficiencies but from the coexistence of divergent perspectives (e.g., Graham et al., 2013). Our findings also suggest that tangible and material concerns triggered by realistic threat perceptions are associated with how individuals reinterpret the situation, which in turn relate to their justice perceptions of sociopolitical events that facilitate or hinder the implementation of responsive measures. Together, these findings indicate that justice operates less as an objective standard for consensus than as a subjective motivation influenced by psychological interpretation, complicating its role in conflict resolution.

Beyond the established roles of RWA and SDO (e.g., Crowson and Brandes, 2017; Dunwoody and Plane, 2019; Forscher and Kteily, 2020; MacWilliams, 2016; Womick et al., 2019), our findings indicate that perceived threat from China indirectly influenced support for Yoon, consistent with media accounts during the impeachment period (see Choe, 2025; Valerio et al., 2025). This pattern parallels evidence from the United States, where support for Trump has been closely associated with perceived outgroup threats involving Mexico, China, Russia, and other regions (see also Dunwoody and Plane, 2019). Authoritarian populist leaders often mobilize support by depicting foreign or migrant outgroups as unjustly harming “virtuous” citizens and by portraying political elites as complicit in this harm (Gelfand and Lorente, 2021; Mols and Jetten, 2016). In South Korea, Yoon similarly identified China as the primary threatening outgroup (Kim, 2024; Park, 2025), repeatedly invoking the rhetoric of “hybrid warfare” throughout the impeachment process. Unlike Trump’s relatively unconstrained unilateralism, however, Yoon operated under geopolitical constraints that required balancing competition with China against reliance on the United States as a security guarantor (Ko, 2006). These findings underscore the importance of perceived outgroup threat—rather than specific policy positions—in shaping support for strong leaders.

The present findings also illuminate the psychological mechanisms underlying belief in electoral fraud and its downstream consequences. Although such beliefs may ostensibly reflect concern for electoral integrity (see Bubeck and Marinov, 2017), from a social psychological perspective they can persist despite institutional improvements when perceived outgroup threats remain salient. In institutional democracies, belief in electoral fraud often involves assumptions of foreign interference (Bubeck and Marinov, 2017; Tomz and Weeks, 2020), as illustrated by U.S. intelligence assessments attributing election interference during Trump’s presidency to Russia (Office of the Director of National Intelligence, 2017; Senate Intelligence Committee, 2018). Our findings indicate that perceived outgroup threats sustain belief in electoral fraud regardless of objective evidence and that such beliefs, in turn, are associated with greater support for strong leaders, which in turn relates to justice perceptions of major sociopolitical events. Beyond its well-documented implications for democratic erosion (e.g., Albertson and Guiler, 2020; Berlinski et al., 2023; Norris, 2019; Thomas et al., 2025), belief in electoral fraud thus appears to function as a mechanism that perpetuates societal polarization and conflict. Notably, this study extends a literature largely centered on the United States by examining electoral fraud beliefs in a non-Western institutional democracy (see also Lee et al., 2024).

Lastly, from an ITT perspective, perceived realistic threats were more strongly associated with support for strong leaders than symbolic threats. Whereas symbolic threats conern challenges to ingroup norms or values (e.g., Biernat et al., 1996; Dunbar et al., 2000; Hainmueller and Hopkins, 2014), realistic threats implicate immediate security and material interests (e.g., Brown et al., 2001; Esses et al., 1998; Zárate et al., 2004). Both symbolic and realistic threats are well known to foster negative outgroup attitudes; however, only realistic threats uniquely predicted heightened support for a strong leader in the Korean context. Theoretically, this asymmetric finding may be explained by the distinct roles of these two types of threat. When people perceive imminent material and tangible threats, they may become more receptive to immediate and exceptional measures implemented by decisive leadership, even legitimizing intergroup violence (Rozmann, 2025). Symbolic threat, in contrast, may not trigger support for such threat-neutralizing actions. This pattern is consistent with authoritarian populist strategies that emphasize material harm inflicted by outgroups and their alleged collusion with political elites (Gelfand and Lorente, 2021; Mols and Jetten, 2016), thereby mobilizing support through perceived competition over limited resources. Our findings align with prior work documenting the impact of realistic threats posed by China (e.g., Ma and Ma, 2023; Maddux et al., 2008) and underscore the theoretical importance of distinguishing threat types, which play distinct roles despite their close association (Riek et al., 2006; Rozmann, 2025). Whereas symbolic threats are associated with attitudes toward Chinese migrants (Hainmueller and Hopkins, 2014), realistic threats more directly account for support for strong leaders who promise to curb intergroup competition. Together, these results extend ITT by highlighting its utility for understanding contemporary authoritarian populism.

### 4.2 Limitations and future directions

Several methodological limitations should be noted. Because realistic threat was conceptualized as perceived economic and resource-related burdens associated with Chinese immigrants, the findings should be interpreted as reflecting this specific dimension of threat rather than broader geopolitical threat perceptions toward China. In addition, while the analyses are consistent with a sequential association, the cross-sectional design does not permit causal inference. Although our sample was drawn from a national online panel, only a small proportion of respondents (75 of 800) reported pro-Yoon support above the scale midpoint, raising concerns about representativeness. Nevertheless, broader inclusion of Yoon supporters would likely strengthen rather than reverse the observed patterns, as our findings align with attitudes documented in media accounts of Yoon’s supporters (see Choe, 2025; Valerio et al., 2025). Media coverage additionally amplifies highly committed supporters; consequently, group norm enforcement and conformity pressures (Kitts, 2003; Morrison and Miller, 2008) may encourage less committed individuals to adopt more rigid positions. Access to pro-Yoon populations is further constrained by politically homogeneous networks (Jost et al., 2018; Blanchar and Norris, 2021) and closed communication channels used to coordinate protest activity and reinforce beliefs in electoral fraud. Because this study prioritized rapid post-impeachment data collection to preserve internal validity, future research should employ targeted sampling and qualitative approaches to better examine variation between Yoon supporters and non-supporters.

This study identifies RWA as an unexpected but crucial predictor of political attitudes and justice perceptions in South Korea than anticipated. RWA—particularly authoritarian submission and aggression—was strongly associated with perceptions of martial law and impeachment, indicating that its role extends beyond that of a control variable. Notably, the divergence in perceived procedural justice may depend on the nature of the political event. Martial law, as a highly abstract and authority-driven measure, may primarily activate authoritarian predispositions, whereas impeachment, as a concrete and leader-directed process, more directly engages support for the political leader. This distinction may account for the stronger role of RWA in evaluations of martial law and of leader support in evaluations of impeachment. In contrast to prior findings, primarily from Western contexts (e.g., Crowson and Brandes, 2017; Forscher and Kteily, 2020; Womick et al., 2019), SDO exerted a comparatively weaker influence, with respondents generally expressing low endorsement. Given South Korea’s geopolitical position—situated among major powers and navigating alliances with dominant countries—Koreans may find it challenging to endorse SDO’s notion that superior groups naturally dominate inferior ones. These results underscore that similarly labeled far-right movements may be underpinned by distinct psychological dynamics across national contexts, highlighting the need for context-sensitive theorizing in South Korean political psychology.

Finally, we call for greater scholarly attention to interventions that leverage justice as a tool for addressing social conflict (see Brockner and Bobocel, 2024; Mikula and Wenzel, 2000; Okimoto and Gollwitzer, 2025). Because polarized groups may be equally motivated by justice yet divided by its subjective interpretation, effective strategies must target the psychological bases of justice perceptions. One possible approach involves targeting perceived realistic threats from China; however, directly dismissing such threats or denying election interference may backfire by increasing the cognitive fluency of existing beliefs and reinforcing them (see Unkelbach et al., 2019). By identifying these underlying mechanisms, future research may inform interventions aimed at alining justice perceptions and facilitate durable cooperation under conditions of uncertainty (Lind and van den Bos, 2002; van den Bos et al., 1997, 2001), thereby helping to mitigate the recurrence of sociopolitical conflict (Mikula and Wenzel, 2000).

## Data Availability

The original contributions presented in the study are publicly available. This data can be found here: Dataset: https://doi.org/10.23668/psycharchives.21851; Questionnaire: https://doi.org/10.23668/psycharchives.21852.
