# Supplementary material for: Realistic threats escalating societal conflict: electoral distrust, strong leader support, and divided justice in South Korea’s 2024–2025 political crisis
Source: Front Psychol. 2026 May 4;17:1808513. doi: 10.3389/fpsyg.2026.1808513 (PMC13180590; doi:10.3389/fpsyg.2026.1808513)
Supplement: Supplementary file 1 [file Supplementary_File_1.DOCX]

|  | Support for Yoon's  Foreign Policies | USA | Japan | China | Russia | North Korea | M(SD) |
| --- | --- | --- | --- | --- | --- | --- | --- |
| Support for Yoon's  Foreign Policies | 1 |  |  |  |  |  | 2.80(1.86) |
| USA | .356******* | 1 |  |  |  |  | 5.19(1.36) |
| Japan | .469******* | .594******* | 1 |  |  |  | 4.23(1.62) |
| China | ‒.295******* | ‒.006 | .035 | 1 |  |  | 4.05(1.51) |
| Russia | ‒.155******* | ‒.038 | .044 | .551******* | 1 |  | 3.73(1.44) |
| North Korea | ‒.403******* | ‒.212******* | ‒.205******* | .492******* | .481******* | 1 | 3.53(1.74) |

**Supplementary Materials**

*Descriptive statistics and correlation matrix showing the associations between support for Yoon’s foreign policies and attitudes toward relations with individual countries. M(SD), Mean (standard deviation).*

******* $p<.001$
